# Lectures on the Operations of Surgery, &c. &c.

**Published:** 1846-03

**Authors:** 


*Lectures on the Operations of Surgery, &c. &c.* By ROBERT LISTON, Esq. F. R. S., &c. &c. With numerous additions, by THOS. D. MUTTER, M. D., Professor of Surgery in Jefferson Medical College, &c. &c. Philadelphia: LEA & BLANCHARD, 1846, pp. 565.

The high character of all of Mr. Liston's writings, and the extensive practical knowledge of the various subjects he treats, render any commendation on our part unnecessary; and the additions by Dr. Mutter render the work still more valuable to the American physician and the dentist, by embodying the improvements and skill of our own surgeons.

